# An old mismanaged Lisfranc injury treated by gradual deformity correction followed by the second-stage internal fixation

**DOI:** 10.1007/s11751-016-0273-3

**Published:** 2017-01-05

**Authors:** Mehraj D. Tantray, Khurshid Kangoo, Asif Nazir, Muzamil Baba, Raja Rameez, Syed Tabish, Syed Shahnawaz

**Affiliations:** Department of Orthopaedics, Bone and Joint Hospital, Barzulla, GMC, Srinagar, Srinagar, Jammu and Kashmir 190005 India

**Keywords:** Lisfranc injury, Ilizarov, Late diagnosis

## Abstract

The Lisfranc fracture-dislocation of the foot is uncommon and diagnosis is often missed. The Lisfranc joint involves the articulation between medial cuneiform and base of the second metatarsal and is considered a keystone to structural integrity to the midfoot. The articulation has a stabilization effect on longitudinal and transverse arches of the foot. A neglected or untreated injury to the Lisfranc joint can lead to secondary arthritis and significant morbidity and disability. We present a case of a neglected Lisfranc fracture-dislocation in a 28-year-old female patient who presented 3 months after injury. A staged treatment of distraction with an Ilizarov ring fixator followed in the second stage by the removal of ring fixator and internal fixation with K wires was performed. There was complete relief of pain and a good functional outcome at 3 months after treatment.

## Introduction

Injuries to the tarsometatarsal joints are not common and represent less than 0.2% of all orthopaedic injuries with a reported incidence of 1 per 55,000 individuals [1]. The injury is commonly missed due to gross swelling masking the deformity and subtle findings on radiological evaluation which requires careful attention. Re-examination after the decrease in oedema for persistent pain and aggravation of pain or instability on stress examination warrants further investigation [2]. The Lisfranc joint injury is notorious for developing secondary arthritis if left untreated or treated with residual incongruity [3].

## Case history

**Fig. 1:**
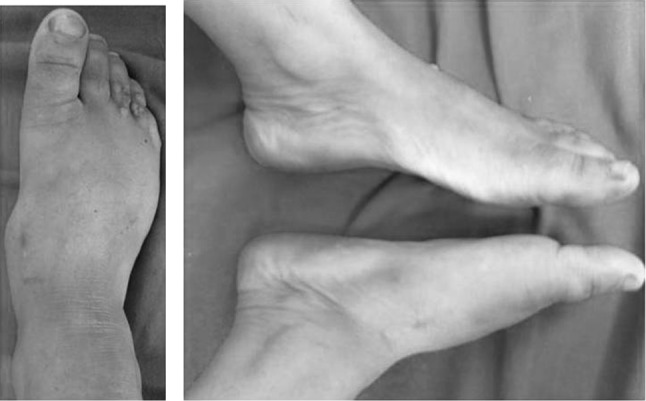
Clinical picture of involved right foot on presentation (uninvolved left foot for comparison)

**Fig. 2:**
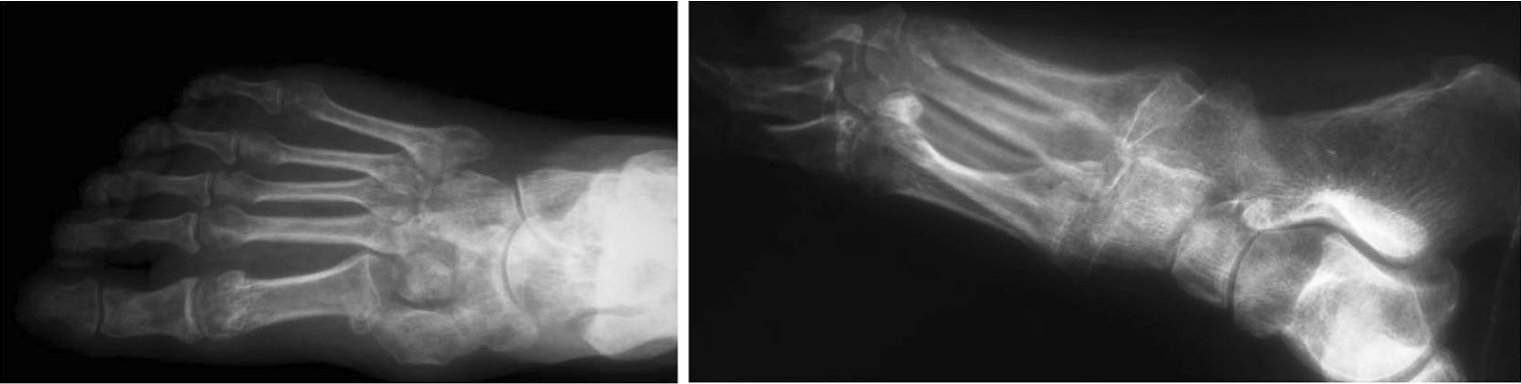
AP and lateral radiograph of involved foot on presentation

**Fig. 3:**
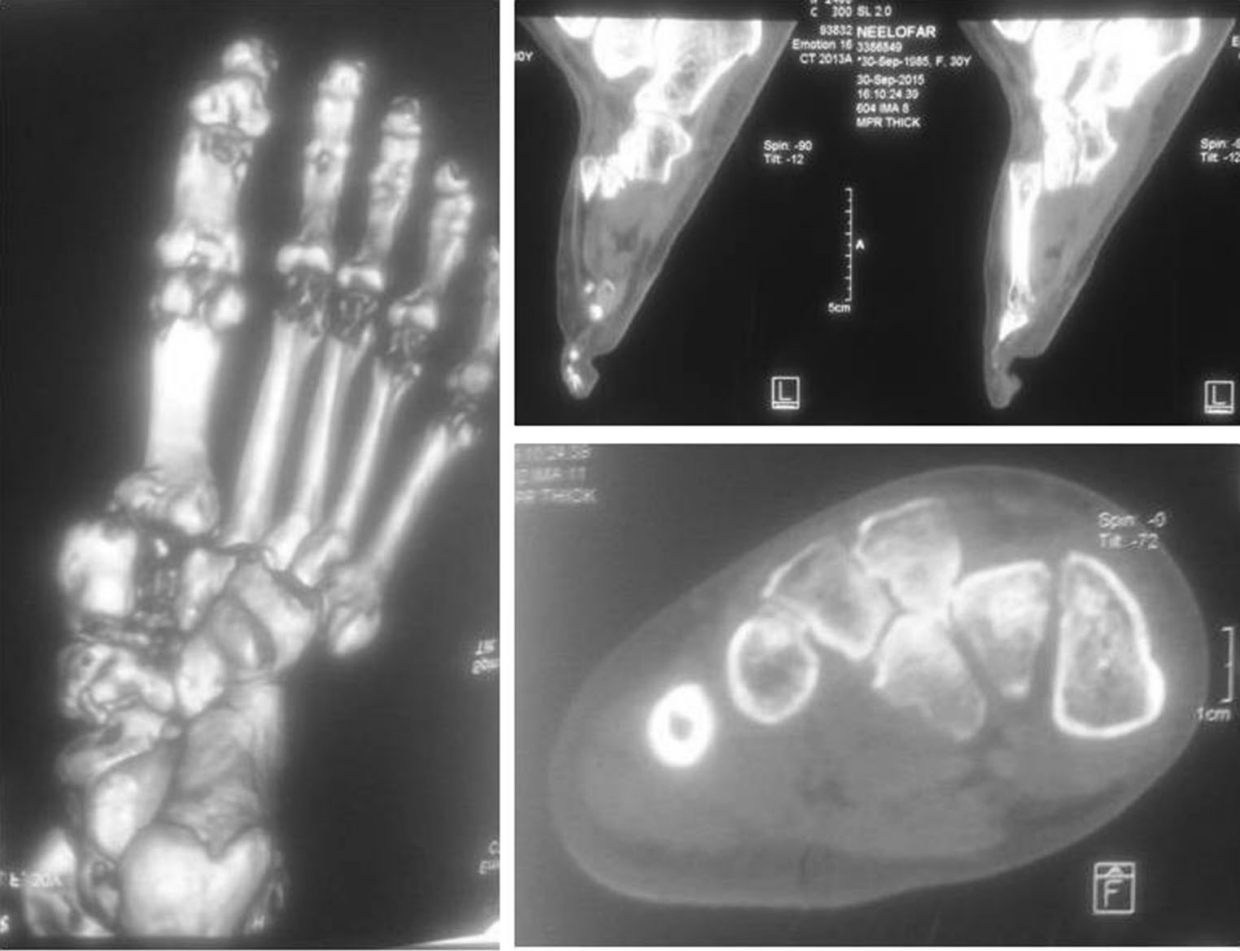
CT images of involved foot on presentation

**Fig. 4:**
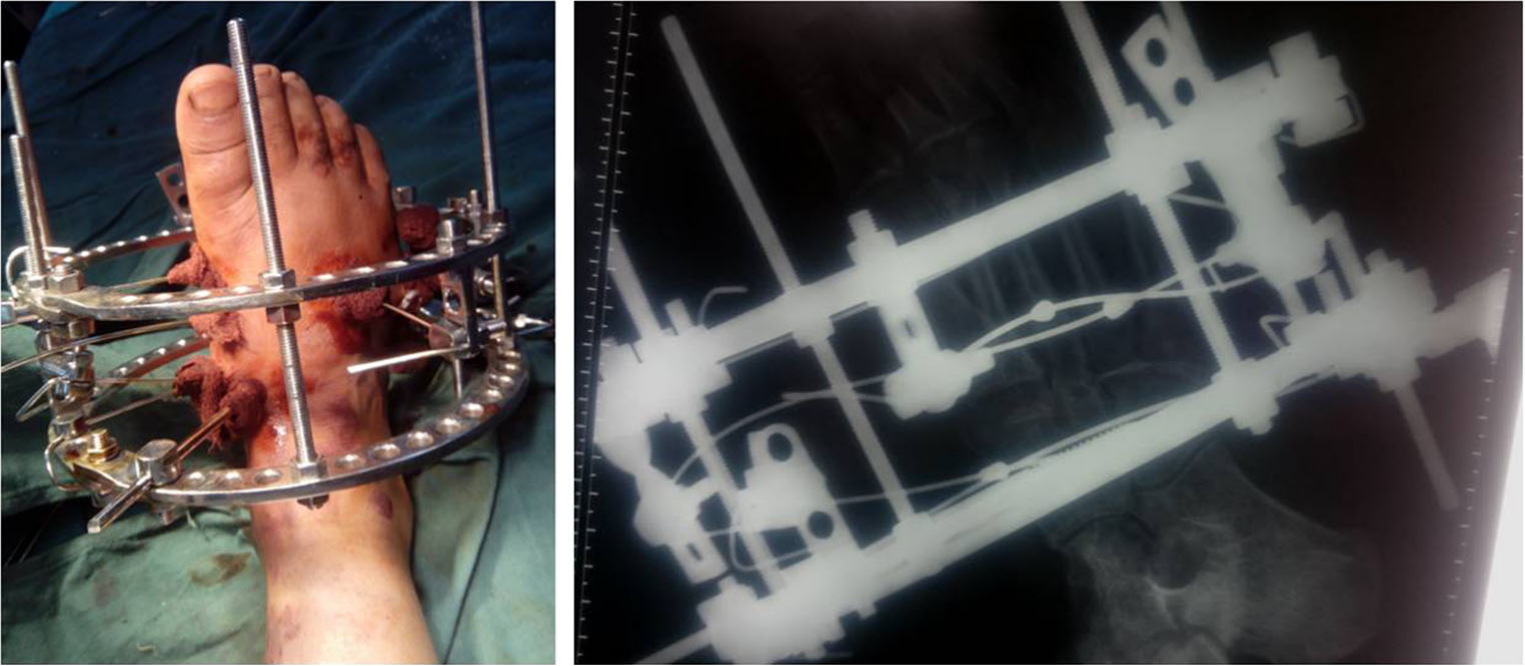
Clinical picture and X-ray on Ilizarov ring fixator

**Fig. 5:**
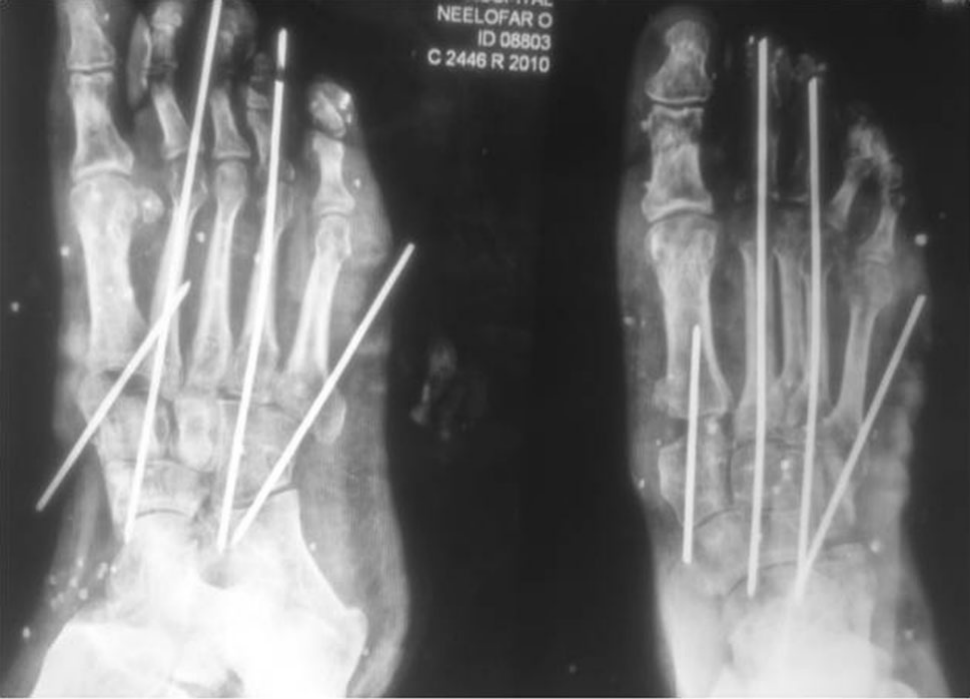
Oblique and AP radiograph of foot after open reduction and K wire fixation

**Fig. 6:**
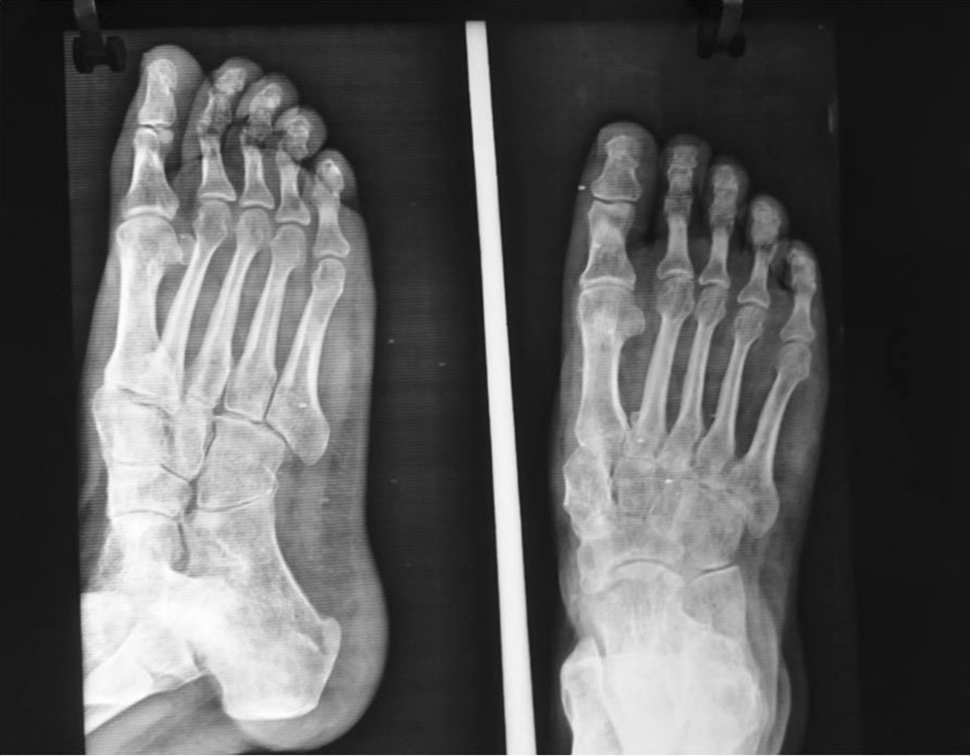
X-rays–AP and oblique view of the foot at 3-month follow-up

**Fig. 7:**
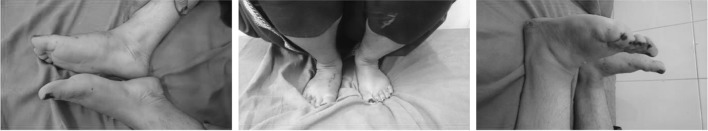
Final clinical picture at 3-month follow-up

A 28-year-old female patient presented with difficulty and pain on walking on her right foot. There was a history of injury to the same foot 12 weeks prior while she was working on a farm. She sought help from a traditional bone setter who performed manipulation and used indigenous herbs and splintage. She was kept non-weight bearing for eight weeks. When the acute pain and swelling had subsided, she noticed a deformity on medial border of foot. She was not able to carry out routine activities and experienced disabling pain on prolonged weight bearing. The examination showed the right foot had a prominence on the medial border and loss of longitudinal arch when compared to opposite foot (Fig. 1). Radiographs of the affected foot showed a Lisfranc fracture-dislocation with lateral and dorsal displacement of all metatarsals with overriding of metatarsals on the tarsus (Figs. 2, 3). In anticipation of the expected difficulty in reducing the tarsometatarsal joints in this patient, a two-stage procedure was planned and discussed with the patient. An Ilizarov ring fixator with the proximal ring holding in midfoot region and distal ring holding the metatarsal region was applied, and gradual distraction at tarsometatarsal joints started 3 days postoperatively for 3 weeks until the tarsometatarsal joints were over-distracted (Fig. 4). In the second stage, the ring fixator was removed and an open reduction and internal fixation with Kirschner wires done with the alignment checked under X-ray image intensification intra-operatively (Fig. 5). Kirschner wires were inserted between the medial cuneiform and the first metatarsal, the intermediate cuneiform and the second metatarsal, the lateral cuneiform and the fourth metatarsal and the fifth metatarsal and cuboid and a short leg cast was applied postoperatively. The K wires were removed after 3 weeks a short leg cast was reapplied and the patient was kept non-weight bearing. This cast was removed at 6 weeks, and weight bearing was commenced (Figs. 6, 7). At a 3-month follow-up, the patient was asymptomatic and had returned to her routine activities without pain.

## Discussion

The French surgeon Jacques Lisfranc de St. Martin reported on midfoot injuries which occurred when cavalrymen fell from their horses with a foot remaining plantar flexed in the stirrup [4]. The Lisfranc complex has both bony and ligamentous structures that provide support to the transverse arch of midfoot. The second metatarsal is locked in between cuneiform bones and adds to the bony stability and ligamentous stability provided by the intercuneiform ligaments [5]. Injuries to the Lisfranc joints are seen commonly in road traffic accidents where the mechanism of injury can be both direct and indirect. In the direct mechanism, there is a crushing injury to the foot with no specific fracture patterns identified where as in the indirect mechanism there is violent plantar flexion or abduction force sustained to foot. During the abduction force, the second metatarsal base gets locked in the cuneiform recess and sustains a fracture (the fleck sign) [6]. As many as 20% of Lisfranc joint injuries are missed upon the initial examination [7]. Diagnosis is made by X-rays of the foot (AP, 30° oblique and lateral views). A high index of suspicion must be maintained for these injuries, and additional imaging—stress radiographs, weight-bearing radiographs, CT or MRI—performed as indicated [8]. On reduction, fixation is done with either screws (cannulated or uncannulated) or Kirschner wires. Screw fixation provides greater stability, but over-compression may cause damage to the joint surfaces. Stabilization with Kirschner wires is simpler and removal easier, but fixation is less stable and there is a risk for pin track infections [9]. Ebraheim et al. have stated that dorsolateral displacement of the second metatarsal base by 1 or 2 mm results in a reduction of the tarsometatarsal articular contact area by 13.1 and by 25.3%, respectively. They have also stated that, regardless of the modality, anatomical alignment needs to be maintained to decrease the risk of posttraumatic arthritis, chronic instability, and pain [10].

In the past, neglected cases of this injury were treated by arthrodesis to achieve a painless and functional foot [10, 11]. A single case of a Lisfranc injury reduced using a Wagner external fixator device and internal fixation using 4-mm cannulated cancellous screws has been reported [12]. In this report, a successful anatomical reduction of the joint was accomplished with prior gradual correction in an Ilizarov fixator and then supplemented with open fixation using Kirschner wires; there was correction of deformity and a painless functional foot at 3 months after treatment.
